# The complete mitochondrial genome of Eurasian Sparrowhawk *Accipiter nisus* (Accipitriformes: Accipitridae)

**DOI:** 10.1080/23802359.2020.1851154

**Published:** 2021-03-11

**Authors:** Ki-Yoon Kim, Yung-Chul Park, Young-Jun Yoon, Kwang-Bae Yoon

**Affiliations:** a Division of Forest Science, College of Forest & Environmental Sciences, Kangwon National University, Chuncheon, Republic of Korea; b Research Center for Endangered Species, National Institute of Ecology, Republic of Korea

**Keywords:** Mitogenome, *Accipiter nisus*, phylogenetic analysis, Accipitridae

## Abstract

The mitogenome of the *Accipiter nisus* is a circular module of 18,352 bp, which consists of 39 genes, containing 2 rRNA genes (12S rRNA and 16S rRNA), 13 protein-coding genes, 22 tRNA genes, and two non-coding regions (control region and pseudo control region). The mitogenome of *A. nisus* is composed of 31.3% A, 25.5% T, 30.4% C, 12.8% G, and 76.3% AT. The phylogenetic analysis revealed that *A. nisus* individuals was well grouped in Accipitridae and more closely related to genus *Circus* than other *Accpiter* species

The Eurasian Sparrowhawk (*Accipiter nisus*) is a small-sized raptor species that hunt small and medium-sized passerines in the forest environment (Rytkönen et al. 1998; Lehikoinen et al. 2010; Hussain et al. 2016). It is distributed in an extremely large range encompassing most of Europe and Asia, and is well adapted to subtropical and temperate areas (Hervías et al. 2017). This species is designated as second-grade endangered species of wild fauna and flora in South Korea (National Institute of Biological Resources 2019).

Muscle tissues of *A. nisus* were collected from the road-killed carcass at Inje-gun, Gangwon-do (38°07′18.4 N″, 128°10′10.2ʺE). A Voucher specimen (KNUWB-04) was deposited in the Wildlife and Fish Conservation Center of the Institute of Forest Science, Kangwon National University. DNeasy Blood & Tissue Kit (Qiagen, Valencia, CA, USA) was used for DNA extraction according to the manufacturer’s instruction. A previously published mitogenome (KM360148) of *A. nisus* (Zhang et al. 2016) was used as a reference for PCR primers and Geneious prime (Kearse et al. 2012) was used for gene annotation and aligned with that of the other 13 Accipitridae species using Clustal W implemented in Geneious Prime (Auckland, New Zealand). The phylogenetic tree was constructed by MEGA-X (Kumar et al. 2018) using the Maximum likelihood (ML) based on the mtREV with frequencies (+F) with gamma distributed with invariant sites (G + I) was selected as the best model of evolution (Jones et al. 1992; Kumar et al. 2018). The Eurasian magpie *pica pica* (ND015200) was used as an outgroup.

The complete mitogenome of *A*. *nisus* (GenBank accession no. MN929010) was 18,352 bp in length and contained two ribosomal RNAs (*12S rRNA* and *16S rRNA*), 13 protein-coding genes (PCGs), 22 transfer RNAs (tRNAs), and two non-coding regions (control region and the pseudo control region).

The base composition for the mitogenome sequence is AT-biased, with nucleotide composition of 31.3% A, 25.5% T, 30.4% C, 12.8% G and 76.3% AT. Length of the two rRNA genes (*12 s rRNA* and *16 s rRNA*) were 971 bp (52.1% A + T) and 1602bp (53.4% A + T), respectively.

The total length of the 13 mitochondrial PCGs of the *A. nisus* is 14,187 bp (57.2% A + T), which encodes 3797 amino acids without stop codons. All protein-coding genes initiate with ATG except COX1 (GTG), ND3 (ATC) and ND5 (ATA). Total 22 tRNA genes, including two leucine-tRNA genes (*tRNA^Leu (CUN)^* and *tRNA^Leu (UUR)^*) and two serine-tRNA genes (*tRNA^Ser (UCN)^* and *tRNA^Ser (AGY)^*), were present in the mitogenome.

Mitochondrial genome of *A. nisus* contains two non-coding regions (D-loop, pseudo control region). D-loop is 2,033 bp in length, located between *tRNA^Thu^* and *tRNA^Pro^*. The Pseudo control region is 760 bp in length, located between *tRNA^Glu^* and *tRNA^Phe^*.

In comparison with the previously published mitogenome of the *A. nisus* (KM360148), which has a total size of 18,647 bp, 99.8% sequence similarity was found in the other regions except for D-loop (96.7%) and pseudo control region (99.7%)

The phylogenetic analysis revealed that *A. nisus* is well placed within the tribe Accipitridae (Figure 1), which forms a sister clade to Chinese *A. nisus* (KM360148). It is analyzed that our sample is more closely related to the genus *Circus* (KT438620, KU237286) than other *Accpiter* species. Earlier datasets have shown genus *Circus* to be sister to the genus *Accpiter* (Barrowclough, 2014), but Oatley et al. (2015) suggest that have no strong support in which lineages are the exact sister-group to the Circus clade.

**Figure 1.**
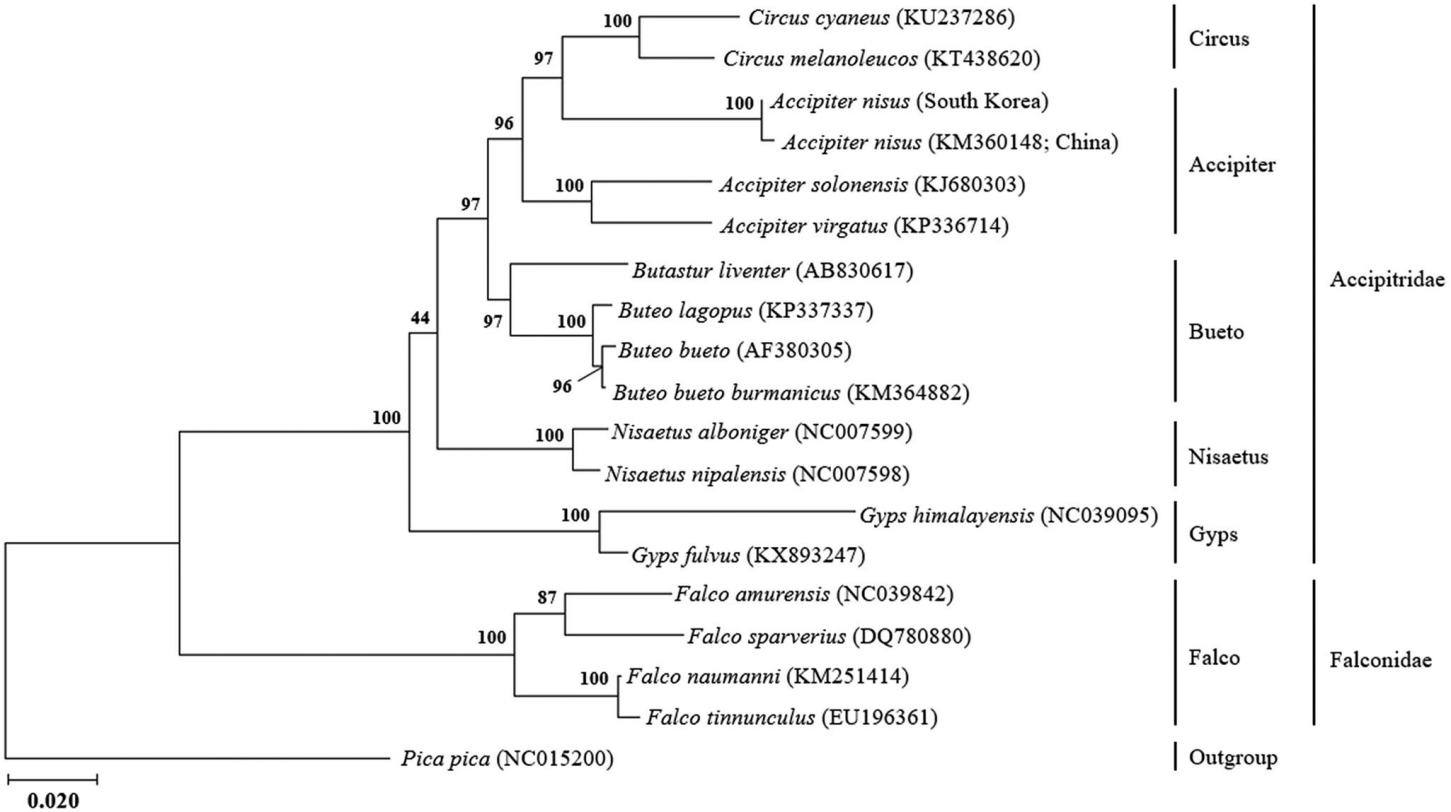
The intraspecific phylogeny of *Accipiter nisus* based on mitogenome sequences. The phylogenetic tree was generated using the maximum likelihood based on mtREV with frequencies (+F) with gamma distributed with invariant sites (G + I). The robustness of the tree was tested with 2000 bootstraps. The numbers on the branches indicate bootstrap values.

## Data Availability

The data that support the findings of this study are openly available in GenBank of NCBI at https://www.ncbi.nlm.nih.gov, reference number MN929010.

## References

[CIT0001] Barrowclough GF, Groth JG, Lai JE, Tsang SM. 2014. The phylogenetic relationships of the endemic genera of Australo-Papuan hawks. J Raptor Res. 48(1):36–43.

[CIT0002] Hervías SP, González YG, Pereira EM, Vulcano A, Cabral RCSR, Coelho NG, Fagundes IC, Castello LB, Nunes MN, Gouveia CA, et al. 2017. The Eurasian Sparrowhawk of Macaronesia (*Accipiter nisus granti*): nesting territories, phenology, and breeding success on Madeira Island, Portugal. J Raptor Res. 51(1):15–24.

[CIT0003] Hussain T, Ashraf I, Ahmed I, Ruby T, Rafay M, Abdullah M, Akhtar S. 2016. Comparison of diet analysis of Eurasian Sparrowhawk, *Accipiter nisus* and Black Kite, *Milvus migrans* (Accipitridae: Accipitriformes) from Southern Punjab, Pakistan. Pak J Zool. 48:789–794.

[CIT0004] Jones DT, Taylor WR, Thornton JM. 1992. The rapid generation of mutation data matrices from protein sequences. Comput Appl Biosci. 8(3):275–282. 1633570 10.1093/bioinformatics/8.3.275

[CIT0005] Kearse M, Moir R, Wilson A, Stones-Havas S, Cheung M, Sturrock S, Buxton S, Cooper A, Markowitz S, Duran C, et al. 2012. Geneious Basic: an integrated and extendable desktop software platform for the organization and analysis of sequence data. Bioinformatics. 28(12):1647–1649. 22543367 10.1093/bioinformatics/bts199 PMC3371832

[CIT0006] Kumar S, Stecher G, Li M, Knyaz C, Tamura K. 2018. MEGA X: molecular evolutionary genetics analysis across computing platforms. Mol Biol Evol. 35(6):1547–1549. 29722887 10.1093/molbev/msy096 PMC5967553

[CIT0007] Lehikoinen A, Saurola P, Byholm P, Lindén A, Valkama J. 2010. Life history events of the Eurasian sparrowhawk *Accipiter nisus* in a changing climate. J Avian Biol. 41(6):627–636.

[CIT0008] National Institute of Biological Resources. 2019. National list of species of Korea; [accessed 2020 Feb 3]. http://kbr.go.kr

[CIT0009] Oatley G, Simmons RE, Fuchs J. 2015. A molecular phylogeny of the harriers (*Circus, Accipitridae*) indicate the role of long distance dispersal and migration in diversification. Mol Phylogenet Evol. 85:150–160. 25701771 10.1016/j.ympev.2015.01.013

[CIT0010] Rytkönen S, Kuokkanen P, Hukkanen M, Huhtala K. 1998. Prey selection by Sparrowhawks *Accipiter nisus* and characteristics of vulnerable prey. Ornis Fenn. 75:77–87.

[CIT0011] Zhang H, Dou H, Yang X, Zhao C, Liu G, Zhang J. 2016. The complete mitochondrial genome sequence of the Sparrowhawk (*Accipiter nisus*). Mitochondrial DNA Part A. 27(3):1648–1649. 10.3109/19401736.2014.958711 25208181

